# Research on neck dissection for oral squamous-cell carcinoma: a bibliometric analysis

**DOI:** 10.1038/s41368-021-00117-5

**Published:** 2021-04-01

**Authors:** Zhou Jiang, Chenzhou Wu, Shoushan Hu, Nailin Liao, Yingzhao Huang, Haoran Ding, Ruohan Li, Yi Li

**Affiliations:** State Key Laboratory of Oral Diseases & National Clinical Research Center for Oral Diseases & Department of Head and Neck Oncology, West China Hospital of Stomatology, Sichuan University, Chengdu, China

**Keywords:** Oral cancer, Cancer therapy

## Abstract

Neck dissection for oral squamous-cell carcinoma (OSCC) is a clinically controversial issue and has therefore been the subject of abundant research. However, no one has performed a bibliometric study on this topic to date. The aim of this study was to assess the development of research on neck dissection for OSCC in terms of the historical evolution, current hotspots and future directions, particularly including research trends and frontiers from 2010 to 2019. Literature records related to research on neck dissection for OSCC were retrieved from the Web of Science Core Collection (WoSCC). CiteSpace was used as a tool to perform a bibliometric analysis of this topic. The survey included 2 096 papers. “Otorhinolaryngology” was the most popular research area. The most active institutions and countries were Memorial Sloan Kettering Cancer Center and the USA, respectively. Shah J.P. was the most cited author. Among the six identified “core journals”, *Head & Neck* ranked first. The top three trending keywords were ‘invasion’, ‘upper aerodigestive’ and ‘negative neck’. ‘D’Cruz AK (2015)’ was the most cited and the strongest burst reference in the last decade. The study evaluated the effect on survival of elective versus therapeutic neck dissection in patients with lateralized early-stage OSCC. The depth of invasion and the management of N0 OSCC were research frontiers in this field. The present study provides a comprehensive bibliometric analysis of research on neck dissection for OSCC, which will assist investigators in exploring potential research directions.

## Introduction

As one of the most common oral malignancies, oral squamous-cell carcinoma (OSCC) accounts for ~4% of all malignant tumours in the body, with over 300 000 newly diagnosed cancer cases throughout the world every year.^1,2^ The status of the regional lymph nodes is considered the most influential prognostic factor for OSCC, as nodal metastasis occurs in over 40% of patients at the time of diagnosis.^3,4^ Worse still, nodal metastasis decreases the survival of patients with OSCC by nearly 50%.^3^ It has been reported that the risk of nodal metastasis depends on multiple complicated factors, such as the size of the primary cancer, location of the tumour, and histopathologic characteristics.^5–7^ Therefore, the management of neck nodal metastasis is one of the most crucial components in the treatment of patients with OSCC.^8^ It was not until the early twentieth century when the first case series of neck dissections was reported by George Crile of the Cleveland Clinic, laying a foundation for the development of cervical lymph-node dissection.^9,10^ To date, several types of neck lymph-node dissections have become applicable, and these include endoscopic assisted selective node dissections and traditional robotic selective node dissections.^11–13^ Despite the marked amount of significant technical progress made in recent decades, there remain some practical challenges in the treatment of OSCC. For instance, for patients with OSCC without clinical neck disease, there is still controversy as to whether it is beneficial to perform selective cervical lymph dissection to eliminate possible occult lymphatic metastasis.^14–16^ Some studies have demonstrated that selective neck dissection is critical for improving the OSCC prognosis given that occult cervical metastasis was found in at least 21% of patients with N0 OSCC.^17–20^ Conversely, no therapeutic benefit is expected to be achieved for patients without neck disease who receive elective neck dissection (END); indeed, they may incur additional morbidity from treatment.^15^ Additionally, close follow-up is recommended for patients with a clinically negative neck (cN0) but without perineural invasion or lymphovascular invasion in the primary tumor.^21^

Bibliometrics is a useful approach to describe the development trend of the research field.^22^ CiteSpace is a tool for visualizing and analysing trends and patterns in scientific papers, and its features help scientific researchers understand network patterns, such as identifying rapidly growing subject areas and finding and tracking citation hotspots.^23,24^ In recent years, many studies have been carried out on neck dissection for OSCC, yet none has employed a bibliometric analysis of this topic. Here, we adopted bibliometric analysis to conduct qualitative and quantitative evaluations of research on neck dissection for OSCC, based on which it is expected that, in this field, emerging trends and hotspots will be identified and future research priorities will be predicted.

## Results

### General analysis

**Fig. 1:**
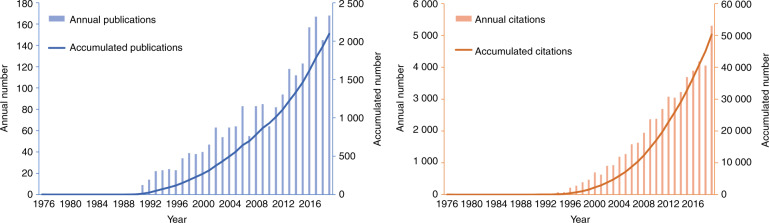
The distribution of publications and their citations from 1976 to 2019. The accumulative numbers of publications and their citations grew slowly in the early stage, whereas an apparent and qualitative leap occurred from 2010 to 2019

A total of 2 096 articles and reviews concerning research on neck dissection for OSCC were selected out of 2 183 literature records retrieved on WoSCC. The time span extended from 1976 to 2019. The first paper was published in 1976, the title of which was *block dissection of the neck for squamous-cell carcinoma of the mouth and lips*, and the first citation was published in 1977. The distribution of publications and their citations is presented in Fig. 1 by year from 1976 to 2019. No significant development or progress was observed in this field during the early stage. The accumulative number of publications did not exceed 500 until 2005, and the accumulative number of citations did not exceed 10 000 until 2007. However, an apparent and qualitative leap of the accumulative numbers occurred from 2010 to 2019, indicating that research on neck dissection for OSCC was becoming a hot and intriguing research topic and that there had been numerous major breakthroughs during this period.

### Category analysis

A total of 18 research areas are represented in the co-occurrence analysis of WOS categories. The majority of publications mainly focused on “otorhinolaryngology” (801), followed by “surgery” (728), “oncology” (581), “dentistry, oral surgery & medicine” (471) and “radiology, nuclear medicine & medical imaging” (147), showing that neck dissection for OSCC is a multifaceted and multidisciplinary field that covers a wide variety of interests. Moreover, the latest research subject categories were “pathology”, “science & technology - other topics” and “multidisciplinary sciences”, all appearing for the first time in 2017.

### Institution and country analysis

The co-operative analysis of institutions showed that Memorial Sloan Kettering Cancer Center was the most active institution by contributing 37 publications and collaborating with 19 other institutions. Based on the highly cited articles from this institution, we found that its research topics mainly lay in the clinical management of cN0 OSCC,^25^ as well as the evaluations of predictors of poor outcomes in OSCC, such as occult nodal metastasis,^26^ lymph-node density,^27^ and primary tumour thickness.^28^

Collaborative studies on neck dissection for OSCC have been performed in 42 countries. Among them, the USA is the most prolific country, with a total of 555 papers published, in a co-operative relationship with 29 other countries, followed by Japan (217), Germany (190), China (141), and England (141). Notably, a sudden increase was observed in the scientific outputs of the USA from 1991 to 1998. A similar phenomenon also occurred in Germany from 2001 to 2004 and in England from 1999 to 2003 but not in Japan or in China. The sudden increase, also known as “burst”, may be the result of breakthrough achievements in this field. In addition, among the top 10 institutions, half are based in the USA, suggesting that the USA not only is actively involved but also plays an important role in research on neck dissection for OSCC.

### Author analysis

The co-operative analysis of authors showed that Remco de Bree was the most active researcher in collaborative studies. In his 11 collaborative publications from 2015 to 2016, the research priority chiefly fell on sentinel node biopsy (SNB) as a reliable and safe oncological technique for staging early-stage cN0 OSCC.^29–31^ However, an author’s influence in a certain scientific field depends more on the number of citations. The co-citation analysis showed that 261 authors were cited 11 138 times in total. Among them, Shah J.P. was the most cited author, with 481 citations, followed by Byers R.M. (464), Woolgar J.A. (384), Yuen P.W. (324), and Spiro R.H. (317). Three of them had a burst citation during different periods, from 1993 to 2001 for Shah J.P., from 1993 to 2002 for Byers R.M., and from 1998 to 2003 for Spiro R.H., while D’Cruz A.K. (202) had the strongest citation burst from 2016 to 2019. It is during or just before these periods that the author made the most prominent achievements.

### Journal analysis

The authority and influence of a journal are mainly measured by the number of citations. The co-citation analysis of journals revealed that a total of 124 journals were cited 22 407 times. Samuel C. Bradford first described how information was scattered for a given field based on the distribution of references in 1934, and he discovered that when equally dividing all references into three zones, the citations for the first zone would come from a small group of “core journals”.^32^ According to Bradford’s law, the core journals in the field of neck dissection for OSCC were identified as follows: *Head and Neck Journal for the Sciences and Specialties of the Head and Neck*, *Laryngoscope*, *Archives of Laryngology*, *the American Journal of Surgery*, *Oral Oncology* and *Cancer*. These journals are a fine choice for relevant researchers to explore literature that interests them or to submit a manuscript.

### Keyword analysis

**Fig. 2:**
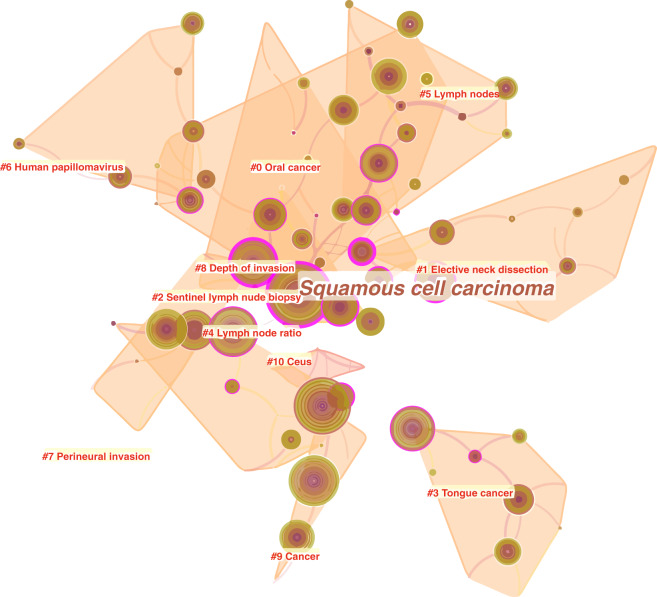
The co-occurrence network of keywords related to research on neck dissection for OSCC from 2010 to 2019. The whole network comprises 100 nodes and 109 links. Each node represents a keyword, and each link between two nodes represents the co-occurrence relationship between two keywords. The size of the node represents the frequency of the keyword. As shown, ‘squamous cell carcinoma’ was the largest node, and its outer purple ring indicated high centrality. All keywords were divided into 11 clusters from #0 to #10 with the respective label, which reflected the topic of the respective cluster

To better illustrate hotspots and frontiers in research on neck dissection for OSCC, we set the time span of the co-occurrence analysis of keywords as the last decade (from 2010 to 2019). The mapping knowledge domain showed 100 nodes (keywords) and 109 links between nodes (Fig. 2), and ‘squamous cell carcinoma’ was the most frequent keyword, alongside a high intermediate centrality (purple ring) of 0.96, which determined its middle position in the mapping knowledge domain. By clustering, a total of 100 keywords were divided into 11 clusters, the labels of which are shown in Fig. 2. For instance, the label of cluster #3 suggested that tongue cancer was a popular research topic of OSCC, which also meant that the tongue was the most common site of OSCC. Furthermore, based on the year in which each keyword first appeared, cluster #8 labelled ‘depth of invasion’ was the latest cluster (mean year = 2014) and best represented the research frontiers in this field. It contained a total of six keywords, the frequency of which from the highest to the lowest were ‘prognosis’, ‘tumour thickness’, ‘invasion’, ‘depth’, ‘depth of invasion’, and ‘rational’.

The occurrence frequency of some keywords was found to be suddenly and drastically increased in a certain period, which was referred to as a “burst”. Burst keywords can well reflect research trends in a scientific field. The top three keywords with the strongest burst were ‘invasion’, ‘upper aerodigestive tract’, and ‘negative neck’ (Table 1).

**Table 1:** The top 10 keywords with the strongest burst on neck dissection research for OSCC from 2010 to 2019

Rank	Keywords	Strength of burst	Beginning year	Ending year
1	Invasion	10.809 2	2017	2019
2	Upper aerodigestive tract	6.996 4	2011	2013
3	Negative neck	6.933 3	2014	2017
4	Stage i	5.909 0	2010	2012
5	Selective neck	5.222 4	2010	2011
6	Mouth	5.156 2	2010	2012
7	Extracapsular spread	5.150 8	2017	2019
8	Reconstruction	5.014 4	2014	2015
9	Transoral robotic surgery	4.865 2	2013	2015
10	Trial	4.786 0	2013	2014

### Reference analysis

**Fig. 3:**
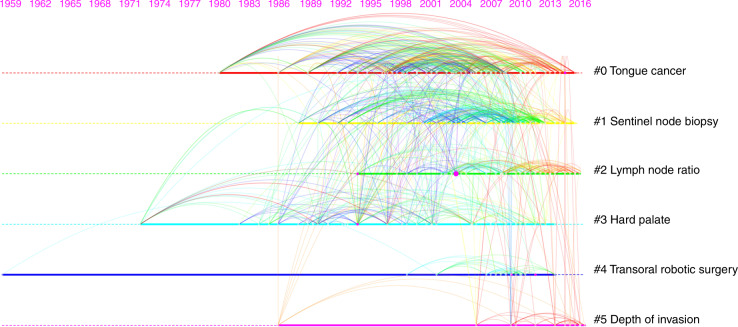
Timeline view of co-cited references related to neck dissection for OSCC from 2010 to 2019. Each node represents a cited reference, and each link between two nodes represents the co-citation relationship between two references. The size of the node represents the citation count of the reference. All references were divided into 6 clusters from #0 to #5 with diverse labels. Based on the publication year of each reference, cluster #5 labelled with ‘depth of invasion’ was the latest (mean year = 2011), reflecting the research trends and frontiers in this field

The 2 096 selected papers were derived from WOS cited references, the cited references of which constitute the network of co-cited references. We analysed the citing references related to research on neck dissection for OSCC in the last decade (from 2010 to 2019), and 10 references with the most citations out of 205 cited references in total are listed below (Table 2). ‘D’Cruz AK (2015)’ was the most cited reference, which was the largest node and was also called the “landmark node” in the network of co-cited references, thus reflecting the research base in this field. By clustering, all cited references were divided into 6 clusters with diverse labels (Fig. 3). According to the publication year of each cited reference, clusters #3 (mean year = 1999), #0 (mean year = 2003), #4 (mean year = 2005), #1 (mean year = 2006), and #2 (mean year = 2008) were older, whereas cluster #5 (mean year = 2011), labelled with ‘depth of invasion’, was the latest. Furthermore, the coverage of citing references that cited the references in cluster #5 is listed below (Table 3); the higher the coverage of the citing reference, the more its study can indicate the frontiers of research on neck dissection for OSCC. In addition, there was also a “burst” in the network of co-cited references. The top 10 references with the strongest citation burst are listed below (Table 4), and ‘D’Cruz AK (2015)’ was also the strongest burst reference, which further proved the vital importance of that particular study in this field.

**Table 2:** The top 10 most cited references in the network of co-cited references

Rank	Reference	Citation count	Journal	IF	Author	Year
1	Elective versus therapeutic neck dissection in node-negative oral cancer	150	*N. Engl. J. Med.*	74.699	Anil K D’Cruz	2015
2	Use of decision analysis in planning a management strategy for the stage N0 neck	117	*Arch. Otolaryngol.*	2.327	M H Weiss	1994
3	Supraomohyoid neck dissection in the treatment of T1/T2 squamous cell carcinoma of oral cavity	107	*Am. J. Surg.*	2.125	J Kligerman	1994
4	Postoperative concurrent radiotherapy and chemotherapy for high-risk squamous-cell carcinoma of the head and neck	105	*N. Engl. J. Med.*	74.699	Jay S Cooper	2004
5	Postoperative irradiation with or without concomitant chemotherapy for locally advanced head and neck cancer	105	*N. Engl. J. Med.*	74.699	Jacques Bernier	2004
6	Sentinel lymph-node biopsy accurately stages the regional lymph nodes for T1–T2 oral squamous-cell carcinomas: results of a prospective multi-institutional trial	100	*J. Clin. Oncol.*	32.956	Francisco J Civantos	2010
7	The patterns of cervical lymph-node metastases from squamous carcinoma of the oral cavity	88	*Cancer*	5.742	J P Shah	1990
8	Prospective randomized study of selective neck dissection versus observation for N0 neck of early tongue carcinoma	87	*Head Neck*	2.538	Anthony Po-Wing Yuen	2009
9	Patterns of cervical lymph-node metastasis from squamous carcinomas of the upper aerodigestive tract	86	*Am. J. Surg.*	2.125	J P Shah	1990
10	Elective versus therapeutic neck dissection in early carcinoma of the oral tongue	83	*Am. J. Surg*.	2.125	A R Fakih	1989

**Table 3:** The top five citing references, which cited the references in the cluster #5 labeled with ‘depth of invasion’ in the reference analysis

Coverage	Citing reference	First Author	Journal	IF	Year
5	Ajcc 8th edition oral cavity squamous-cell carcinoma staging—is it an improvement on the ajcc 7th edition?	Pollaers, Katherine	*Oral Oncol*.	3.979	2018
4	Intraoperative depth of invasion is accurate in early-stage oral cavity squamous-cell carcinoma	Moe, Justine	*J. Oral. Maxillofac. Surg.*	1.642	2019
4	Depth of invasion in patients with early-stage oral cancer staged by sentinel node biopsy	den Toom, Inne J	*Head Neck*	2.538	2019
4	Elective neck dissection in oral squamous-cell carcinoma: past, present and future	de Bree, Remco	*Oral Oncol*.	3.979	2019
4	Comparison of the 7th and 8th edition american joint committee on cancer oral cavity staging systems	Cramer, John D	*Laryngoscope*	2.456	2018

**Table 4:** The top 10 burst references in the network of co-cited references

Rank	Reference	Burst	Journal	IF	Author	Year
1	Elective versus Therapeutic Neck Dissection in Node-Negative Oral Cancer	28.95	*N. Engl. J. Med*.	74.699	Anil K D’Cruz	2015
2	Sentinel European Node Trial (SENT): 3-year results of sentinel node biopsy in oral cancer	12.17	*Eur. J. Cancer*	7.275	Clare Schilling	2015
3	Early-stage squamous-cell cancer of the oral tongue–clinicopathologic features affecting outcome	9.97	*Cancer*	5.742	Ian Ganly	2012
4	Sentinel node biopsy in head and neck cancer: preliminary results of a multicenter trial	9.61	*Ann. Surg. Oncol.*	4.061	Gary L Ross	2004
5	Can we detect or predict the presence of occult nodal metastases in patients with squamous carcinoma of the oral tongue?	8.52	*Head Neck*	2.538	R M Byers	1998
6	Sentinel node biopsy for early oral and oropharyngeal squamous-cell carcinoma	8.35	*Eur. Arch. Otorhinolaryngol.*	1.809	Sandro J Stoeckli	2009
7	Lymph-node density in oral cavity cancer: results of the International Consortium for Outcomes Research	7.92	*Br. J. Cancer*	5.791	S G Patel	2013
8	Predictive factors of occult metastasis and prognosis of clinical stages I and II squamous-cell carcinoma of the tongue and floor of the mouth	7.86	*Oral Oncol*.	3.979	Tânia Mara Pimenta Amaral	2004
9	The accuracy of head and neck carcinoma sentinel lymph-node biopsy in the clinically N0 neck	7.08	*Cancer*	5.742	T Shoaib	2001
10	Elective neck dissection in oral carcinoma: a critical review of the evidence	6.85	*Acta Otorhinolaryngol. Ital.*	1.51	L P Kowalski	2007

### Trends and frontiers

Of note, the keyword analysis suggested that the latest cluster was labelled ‘depth of invasion’ and that the top three keywords with the strongest burst were ‘invasion’, ‘upper aerodigestive tract’ and ‘negative neck’, with both results reflecting the research trends and frontiers in the field of neck dissection for OSCC in the last decade. In addition, the reference analysis also suggested that the latest cluster was labelled ‘depth of invasion’. The keyword ‘upper aerodigestive tract’ is not specific to OSCC. Therefore, we analysed the top 10 citing references with the most citations that cited these three keywords and conducted a literature review of them as follows.

#### Depth of invasion (DOI)

To more accurately assess tumour behaviour and guide clinical decision making, the eighth edition of the American Joint Committee on Cancer Staging Manual (AJCC 8th) incorporated the clinical as well as pathological DOI into the definition of the pathological T stage of OSCC, which required surgeons to carefully distinguish it from tumour thickness.^33,34^ Furthermore, according to the DOI, tumours are classified as less invasive, moderately invasive, and deeply invasive.^34^ Margaret M Kozak et al. demonstrated that although a greater DOI was associated with worse progression-free survival (PFS) and overall survival (OS) in patients with low-risk, early-stage OSCC, a DOI greater than 4 mm alone did not indicate an increased incidence of relapse in patients treated with surgery alone.^35^ Moreover, in the absence of other adverse pathological features, DOI should not be an independent indication for postoperative radiotherapy in small OSCC.^36^ With regard to the implementation of END, Akira Baba et al. reported that oral tongue cancer undetectable on magnetic resonance imaging (MRI) indicated a high possibility of pathological DOI smaller than 4 mm, which can be used as a criterion to avoid unnecessary END in node-negative patients.^37^ Nevertheless, MRI evidence of invasion to the styloglossus and hyoglossus muscles corresponds to a DOI greater than 4 mm, which can be a criterion for the advisability of END.^38^

#### Invasion

This is a significant histopathological parameter that can predict occult cervical lymph-node metastasis in patients with OSCC. It has been reported that blood vessel invasion,^39^ lymphatic invasion,^39^ and muscle invasion^40^ are predictive factors for neck recurrence in early oral tongue carcinoma, suggesting that END may be warranted for patients with these findings. Furthermore, L.X. Niu et al. reported that perineural invasion also serves as a predictive factor for the 5-year OS of squamous-cell carcinoma of the mandibular gingiva.^41^ Recently, Aditi Arora et al. developed a new outcome prediction model for early-stage OSCC based on histopathological parameters with multivariate analysis, namely, the Aditi-Nuzhat Lymph-node prediction score (ANLPS) system.^42^ Patterns of invasion, lymphovascular invasion and perineural invasion were included in this system.

#### Negative neck

Occult cervical lymph-node metastasis was found in at least 21% of patients with cN0 OSCC. Sara Abu-Ghanem et al. demonstrated that END can significantly reduce the risk of regional nodal recurrence and improve disease-specific survival in patients with early-stage cT1-T2N0 oral tongue squamous-cell carcinoma.^43^ With respect to late-stage OSCC, lower regional recurrence rates and improved survival outcomes were observed as the lymph node yield increased for advanced T classification pathological N0 OSCC.^44^ However, Man Ki Chung recommended that SNB be used to replace the current END policy for carefully selected patients with cN0 OSCC since it provides acceptable oncological outcomes by long-term observation, maintaining high accuracy rates through the validation and application phases.^45^ In addition, a meta-analysis suggested that asymptomatic lymph nodes may be left untreated, kept under close observation, and treated once metastasis develops in primary cN0 lip SCC.^46^

On the other hand, the reference analysis showed that ‘D’Cruz AK (2015)’ was not only the most co-cited but also the strongest burst reference in the last decade, indicating its vital importance in research on neck dissection for OSCC. The study evaluated the effect on survival of elective versus therapeutic neck dissection in patients with lateralized stage T1 or T2 OSCC and ultimately concluded that END led to a higher rate of OS and DFS in patients with early-stage OSCC.^19^ The frequency of END has increased overall over the last decade and it has appeared in 107 scientific records, the top 5 of which are shown in Table 5.

**Table 5:** The top five citing references from 2010 to 2019 with most citations that cited END

Rank	Citing reference	Citation count	Journal	IF	Author	Year
1	Early-stage squamous-cell cancer of the oral tongue–clinicopathologic features affecting outcome	132	*Cancer*	5.742	Ian Ganly	2012
2	A meta-analysis of the randomized controlled trials on elective neck dissection versus therapeutic neck dissection in oral cavity cancers with clinically node-negative neck	97	*Oral Oncol.*	3.979	Ayotunde J Fasunla	2011
3	Comparison of morbidity between sentinel node biopsy and elective neck dissection for treatment of the n0 neck in patients with oral squamous-cell carcinoma	96	*Head Neck*	2.538	Karin Murer	2011
4	Sentinel node biopsy for squamous-cell carcinoma of the oral cavity and oropharynx: a diagnostic meta-analysis	77	*Oral Oncol.*	3.979	Tim M Govers	2013
5	Prognostic value of lymph-node density in node-positive patients with oral squamous-cell carcinoma	65	*Ann. Surg. Oncol.*	4.061	Sang Yoon Kim	2011

## Discussion and conclusion

In the field of neck dissection for OSCC, early-stage research developed and progressed very slowly, whereas a remarkable and qualitative leap was observed from 2010 to 2019. Hence, we particularly paid attention to the keywords and citing references over the last decade and discovered that future research directions in this field mainly focused on three aspects: (1) the exploration of the predictive, prognostic, and discriminatory ability of the AJCC 8th staging system for oral cavity cancer and the comparison between the AJCC 8th and previously used AJCC 7th staging systems;^47,48^ (2) the investigation of DOI as a clinical tool to predict occult nodal metastasis and to determine the need for END for early-stage cN0 OSCC;^49,50^ (3) the development and implementation of models and decision support systems that can serve to optimize choices depending on the individual, institutional, population, and other relevant variables to optimize the management of the cN0.^14^

Although there are some limitations in this bibliometric study, for instance, the papers of highly cited authors cannot be traced in the co-citation analysis of cited authors, this study will greatly help relevant researchers comprehend the research development, hotspots, trends, and frontiers of neck dissection for OSCC and determine what remains to be investigated further.

## Methods

All of the original data were obtained from the WoSCC on July 10, 2020. The retrieval strategy used in this study was “TS = (oral or oral cavity or mouth or tongue or gingiva or buccal or palate or floor of mouth) AND TS = (neck dissection and squamous cell carcinoma)”. The time span was set between 1900 and 2019. Languages and document types were set to “all”. The retrieval resulted in 2 183 literature records, all of which were downloaded and exported into CiteSpace for further bibliometric analysis. “Plain Text” was chosen as the file format, and “Full Record and Cited References” was chosen for the record content to obtain all the necessary information. After removing duplicates and selecting document types (articles and reviews) using CiteSpace, we retained 2 096 papers for further bibliometric analysis.

To evaluate the research history, development, hotspots, trends, and frontiers of neck dissection of OSCC, three main types of analyses were performed by CiteSpace: the co-operation, co-occurrence and co-citation analysis. Co-operation refers to the fact that a publication is the scientific output of a joint study by two or more institutions or countries. Multiple keywords listed in one publication constitute a co-occurrence relationship. In addition, if two papers are cited by other papers at the same time, then the two form a co-citation relationship, showing that there may be a certain correlation between their studies.^51,52^

The subjects of this study included categories, institutions, countries, journals, authors, keywords and references of the screened 2 096 publications. The top 50 levels of the most cited or occurring items from each slice (1 year per slice) were selected for analysis, and each level may include multiple qualified nodes. The time period was set from 1976 to 2019, but as we dug into the current research hotspots and future research trends, we focused more on the scientific literature in the last decade (from 2010 to 2019), for instance, keyword analysis and reference analysis. The rest of the parameters and values were default by the software.

The results were presented as visual mapping knowledge domains, such as a cluster network consisting of nodes and links, for easy viewing.^53^ A node represents an item, such as an author, a keyword, a reference, etc., while a link indicates the relationship between nodes, for instance, the co-operation, co-occurrence, or co-citation relationship. Every cluster can be labelled with a term that is extracted from the selected scientific literature. In some cases, the cluster network can be pruned to reduce the number of redundant links and retain the most salient links.
